# Food Insecurity and Cognitive Trajectories in Community-Dwelling Medicare Beneficiaries 65 Years and Older

**DOI:** 10.1001/jamanetworkopen.2023.4674

**Published:** 2023-03-24

**Authors:** Boeun Kim, Laura J. Samuel, Roland J. Thorpe, Deidra C. Crews, Sarah L. Szanton

**Affiliations:** 1 School of Nursing, Johns Hopkins University, Baltimore, Maryland; 2 Johns Hopkins Alzheimer's Disease Resource Center for Minority Aging Research, Bloomberg School of Public Health, Johns Hopkins University, Baltimore, Maryland; 3 Division of Nephrology, Department of Medicine, School of Medicine, Johns Hopkins University, Baltimore, Maryland

## Abstract

**Importance:**

Food insecurity has a known association with prevalent impaired cognition. However, it is unknown whether food insecurity has a longitudinal association with cognitive decline among older adults.

**Objective:**

To determine whether food insecurity is associated with a faster decline in cognitive function among community-dwelling older adults.

**Design, Setting, and Participants:**

This retrospective cohort study used data from a nationally representative sample of Medicare beneficiaries 65 years and older recruited for the National Health and Aging Trends Study (NHATS). Community-dwelling NHATS participants were followed up for a maximum of 7 years (mean [SD] follow-up duration, 5.4 [1.1] years). Data were collected from January 1, 2012, to December 31, 2020, and analyzed from December 23, 2021, to December 6, 2022.

**Exposures:**

Self-reported food insecurity assessed from 2012 to 2019.

**Main Outcomes and Measures:**

Primary outcomes were immediate memory, delayed memory, and executive function collected from 2013 to 2020. Immediate and delayed recall were assessed by a 10-item word-list memory task (range, 0-10, with higher scores indicating more words recalled). Executive function was measured by the clock drawing test (range, 0-5, with higher scores indicating more accurate depiction of a clock). Each year’s cognitive functions were linked to the prior year’s food insecurity data. Linear mixed-effects models with random slopes and intercepts were used to examine the association between food insecurity and cognitive decline. Analytic weights in each year were applied to represent community-dwelling Medicare beneficiaries 65 years and older in 2011.

**Results:**

Of 3037 participants, a weighted 57.8% (raw count, 1345) were younger than 75 years, 56.2% (raw count, 1777) were women, and most (84.9% [raw count, 2268]) were White. Over 7 years, 417 (weighted proportion, 12.1%) experienced food insecurity at least once. Food insecurity was associated with a faster decline in executive function in a fully adjusted model: the mean difference of annual change in executive function score between people exposed to and not exposed to food insecurity was −0.04 (95% CI, −0.09 to −0.003) points. However, food insecurity was not associated with changes in immediate and delayed memory (0.01 [95% CI, −0.05 to 0.08] and −0.01 [95% CI, −0.08 to 0.06], respectively).

**Conclusions and Relevance:**

Among community-dwelling older adults, food insecurity was prevalent and associated with a decline in executive function. Interventions and policies aiming to increase healthy food access or reduce food insecurity should be assessed for their impact on older adults’ cognitive outcome.

## Key Points

**Question:**

Is food insecurity associated with faster cognitive declines among community-dwelling older adults?

**Findings:**

In this cohort study using data from 3037 community-dwelling Medicare beneficiaries 65 years and older, food insecurity reported in late life was associated with a faster decline in executive function.

**Meaning:**

These findings suggest that older adults who report food insecurity may be at higher risk of accelerated decline in executive function; intervention studies are needed to determine whether food assistance programs addressing food insecurity can prevent and/or delay executive function decline among older adults.

## Teaser

This cohort study examines the association of time-variant measures of food insecurity with changes in cognitive function among a nationally representative sample of US adults 65 years and older.

## Introduction

Food insecurity is defined as uncertain access to or inability to acquire nutritionally adequate foods in socially acceptable ways.^1^ An estimated 5.2 million older US residents (6.8% of adults older than 60 years) experienced food insecurity in 2020.^2^ Some groups of older adults are disproportionately affected.^2,3^ For instance, in 2020, 26.5% of older adults with incomes below the poverty threshold and 19.1% of self-identified Black older adults were food insecure, while only 2.9% of older adults with incomes above 200% of the federal poverty line and 5.2% of White older adults were food insecure.^2^ The number of US adults 60 years and older experiencing food insecurity has more than doubled since 2007.^2,4^

Food insecurity adversely affects mental and physical health, including cognitive performance.^5,6,7,8,9^ Because poor cognitive performance can impair quality of life and functional independence in late life, identifying modifiable factors that influence cognitive decline, like food insecurity, has caught the attention of many health researchers and policy makers.^10,11,12^ Food insecurity could be targeted to prevent or delay cognitive decline,^8,12,13^ but associations between food insecurity and cognitive function are underexamined. Determining whether food insecurity is associated with cognitive decline could advance understanding of the role of interventions and policies addressing food insecurity.

Extant literature cannot fully explain the longitudinal impact of food insecurity on cognitive function in older adults due to the nature of a cross-sectional study^13,14^ or a regional cohort study.^15^ Important gaps in this topic area remain. First, food insecurity status likely changes, and it is important to understand the total exposure of food insecurity over time to better estimate the health effect. However, no longitudinal study assessed food insecurity status over time. Second, cognitive trajectories should be followed up more than twice to describe the shape and to distinguish true decline from measurement error.^16^ Third, to our knowledge, no longitudinal study has used data from a nationally representative sample of US older adults, which can limit generalizability of findings.

Accordingly, we undertook a longitudinal analysis to examine the association between time-variant measures of food insecurity with changes in cognitive function among a nationally representative sample of US adults 65 years and older. We hypothesized that food insecurity would be associated with a faster rate of cognitive decline among older adults.

## Methods

### Study Design and Participants

This retrospective cohort study used the existing data set of the National Health and Aging Trends Study (NHATS), a nationally representative cohort of US Medicare beneficiaries 65 years and older. NHATS began participant recruitment in 2011 and replenished in 2015. We used data from the 2011 cohort, and its response rate at initial recruitment was 71%.^17^ Enrolled participants underwent detailed in-person interviews annually, except the 2020 interview conducted by telephone due to the COVID-19 pandemic. We linked each year’s cognitive function assessment to the 1-year prior food insecurity measurement to ensure that the exposure preceded the outcome.^16^ Food insecurity was first assessed in 2012; therefore, we used food insecurity data collected from January 1, 2012, to December 31, 2019, and cognitive function data collected from January 1, 2013, to December 31, 2020. All participants provided informed consent for their participation. These analyses were regarded as exempt by the Johns Hopkins School of Medicine Institutional Review Board because only publicly available data were used. This cohort study followed the Strengthening the Reporting of Observational Studies in Epidemiology (STROBE) reporting guideline.

Of the 8245 individuals enrolled in 2011, 5799 (70.3%) completed the 2013 interview. Of those, we included only community-dwelling individuals (n = 4508), because the food insecurity measure has not been validated among older adults living in residential care, and institutionalized older adults may have qualitatively different experiences of food insecurity. Of those, participants with dementia (n = 245) or mild cognitive impairment (n = 348) at baseline and having only 1 valid cognitive assessment data over the study period (n = 877) were excluded, leaving an analytic sample size of 3037 participants (Figure).

**Figure.**
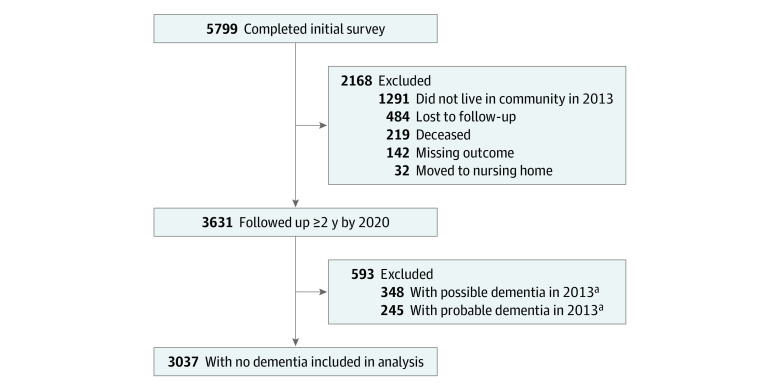
Flowchart of Eligible Participants and Reasons for Dropping Out National Health and Aging Trends Study participants were included if they lived in community settings at baseline, had at least 2 outcome measures over the study duration, and did not have dementia (probable dementia) or mild cognitive impairment (possible dementia) at baseline. ^a^Data were missing for 1 patient.

### Primary Outcomes

The primary outcomes were executive function, immediate memory, and delayed memory scores. Executive function was assessed by the clock drawing test.^18^ Participants were instructed to draw a clock with a specific time and were scored on a scale from 0 (not recognizable as a clock) to 5 (an accurate depiction of a clock) by trained coders.^19^ The interview was administered by telephone in 2020. Previous research^20^ showed that there were no significant differences in mean cognitive scores by test administration mode (telephone vs in person). Memory was assessed by performance on word list memory and recall tests.^21^ Immediate and delayed recall scores were constructed by the total number of words correctly recalled at a given time (immediately and after 5 minutes, respectively), ranging from 0 to 10, where higher scores indicated more words recalled.^19^ The memory recall tests have been validated in telephone interviews.^22^

### Primary Exposure and Adjustment Variables

Food insecurity was measured based on the following 5 items^23^: (1) going without groceries due to limited ability or social support, (2) going without hot meals related to functional limitation or no help, (3) going without eating because of inability to feed oneself or no available support, (4) skipping meals due to insufficient food or money, and (5) days of skipped meals. Participants were classified as food insecure if the summed score was 1 or greater than 1.^23^ This measure is more holistic than other food insecurity measures that focus on only financial constraints because it incorporates risk factors for food insecurity among older adults such as social isolation and reduced mobility in addition to financial strain.^23^

Potential confounders in these analyses were educational level, sex, self-reported race and ethnicity (American Indian or Alaska Native, Asian, Black, Hispanic, Native Hawaiian or other Pacific Islander, and White), age, depression (measured by the Patient Health Questionnaire),^24^ functional disability,^25^ and social isolation scores^26^ at baseline. Race and ethnicity data were included in this model because racial and ethnic minority groups were more likely to experience food insecurity as well as have worse cognitive health. We additionally adjusted for marital status, income, body mass index (BMI; calculated as weight in kilograms divided by height in meters squared), and rural or urban residence as time-varying covariates. Income was reported in 2011, 2013, 2015, 2017, and 2019. Marital status and BMI were measured between 2012 and 2019, and residential areas were extracted from 2012 to 2018 data (eMethods in Supplement 1). Year since study enrollment was also included in these analyses as a time metric.

### Statistical Analysis

Data were analyzed from December 23, 2021, to December 6, 2022. In NHATS, income data were missing for 31% of participants in 2011, 25% in 2015, and 13% in 2019. NHATS imputed income based on reported bracketed income value, sources of income, their amounts, and other auxiliary variables such as home ownership and labor force status using a hot-deck procedure.^27^ Based on a missing-at-random assumption, we replaced the missing values with the imputed income. All other variables were missing at less than 5%. Baseline characteristics were compared between individuals who were food insecure at least once and those who were never food insecure during the study period using Pearson χ^2^ statistics with corrected SE based on sampling design. We used mixed-effects linear models to assess the association of food insecurity with change in cognitive function. Both outcomes and exposure were treated as time-varying variables. One-year time-lagged analyses linked each year’s outcomes to prior exposure data to diminish the possibility of reciprocal causation.^16^ A model for each cognitive function measure included terms for food insecurity, time (years since baseline), and their interaction. There were no nonlinear time trends in cognitive function over the study period. The hypothesis was tested using the interaction term between food insecurity and time, which describes differences in the yearly change in cognitive function comparing participants with vs without food insecurity. All models adjusted for age, sex, race and ethnicity, educational level, depression, functional disability, and social isolation at baseline, which were known to be associated with food insecurity^9,23^ and cognitive function.^28,29,30^ We additionally adjusted for income, marital status, BMI, and residential area over the study period (eFigure in Supplement 1). Adjusted models included statistically significant interactions with time for race and ethnicity, educational level, and age at baseline to account for differential decline in cognitive function scores over time depending on the baseline characteristics. Intercepts and slopes of cognitive trajectory were allowed to vary for each individual as random effects. We applied analytic weights each year to account for complex sampling design and nonresponse, allowing us to generalize the findings to the 2011 Medicare population 65 years and older. To account for potential differences in measurement modality during the pandemic, we conducted a sensitivity analysis to see whether the estimates were changed when data collected in 2020 were excluded. We also performed other sensitivity analyses using marginal structural models with stabilized inverse propensity weights for better adjustment of potential time-varying confounders.^31^ Statistical significance was presumed at 2-sided *P* < .05. Analyses were performed with Stata, version 16.1 (StataCorp LLC) using a module for survey data analyses with multilevel mixed-effects generalized linear models.

## Results

### Demographic Characteristics

The analytic sample consisted of 3037 participants; 1345 (weighted proportion, 57.8%) were younger than 75 years, 1777 (weighted proportion, 56.2%) were women, and 1260 (weighted proportion, 43.8%) were men. A total of 568 participants (7.3%) were Black, 128 (5.1%) were Hispanic, 2268 (84.2%) were White, and 53 (2.5%) were of other race or ethnicity (American Indian or Alaska Native, Asian, and Native Hawaiian or other Pacific Islander). The mean (SD) duration of follow-up was 5.4 (1.1) years, with a maximum duration of 7 years. The baseline weighted prevalence of food insecurity was 2.5% (raw count, 81 participants). Over 7 years, 12.1% of the participants experienced food insecurity at least once (raw count, 417 participants). Participants who experienced any food insecurity over the study interval were more likely to be older, female, part of racial and ethnic minority groups, not living with a partner, and obese and to have lower income, lower educational attainment, depressive symptoms, social isolation, and disability compared with their peers without any food insecurity (Table 1).

**Table 1.** Baseline Characteristics According to Food Insecurity by 2020 in National Health and Aging Trends Study Participants Included in the Analyses^a^

Characteristic	Participant group (N = 3037)		*P* value^b^
	Always food secure (n = 2620)	Any food insecurity (n = 417)	
Age, y			
65-69	538 (29.3)	58 (20.0)	<.001
70-74	655 (29.9)	94 (27.3)	
75-79	572 (20.2)	99 (24.0)	
80-84	496 (13.0)	80 (15.2)	
≥85	359 (7.7)	86 (13.5)	
Sex			
Men	1151 (46.0)	109 (27.7)	<.001
Women	1469 (54.0)	308 (72.3)	
Race and ethnicity			
Black	474 (7.0)	94 (10.0)	.02
Hispanic	106 (4.9)	22 (7.1)	
White	1982 (85.1)	286 (77.7)	
Other^c^	44 (2.4)	9 (3.4)	
Missing	14 (0.7)	6 (1.9)	
Income, $			
0-18 000	648 (19.9)	173 (39.8)	<.001
18 001-41 000	861 (31.7)	148 (33.3)	
>41 000	1111 (48.4)	96 (26.9)	
Educational level			
Less than high school	460 (14.7)	96 (18.9)	.05
High school	730 (27.4)	97 (22.9)	
More than high school	1418 (57.3)	220 (56.5)	
Missing	212 (0.7)	4 (1.7)	
Marital status			
Married or living with a partner	1502 (63.7)	157 (41.5)	<.001
Separated, divorced, widowed, or never married	1118 (36.3)	260 (58.6)	
Depressive symptoms present^d^	221 (7.7)	73 (16.0)	<.001
Social isolation			
Severely isolated	87 (2.9)	32 (9.1)	<.001
Isolated	471 (17.7)	89 (21.4)	
Integrated	2062 (79.4)	296 (69.5)	
No. of disabilities			
0	2414 (93.3)	337 (81.7)	<.001
1-2	168 (5.7)	65 (14.9)	
3-6	38 (1.1)	15 (3.4)	
BMI			
Underweight	33 (1.1)	10 (2.5)	.03
Normal weight	764 (28.5)	134 (31.8)	
Overweight	1043 (40.1)	140 (32.6)	
Obesity	742 (28.82)	127 (32.3)	
Missing	38 (1.4)	6 (0.9)	
Residential area			
Metropolitan	2073 (81.2)	347 (83.6)	.33
Nonmetropolitan	547 (18.8)	70 (16.5)	

Abbreviation: BMI, body mass index (calculated as weight in kilograms divided by height in meters squared).

^a^ Data are presented as No. (%) of participants. Percentages are weighted and were estimated using 2013 sampling weight to represent the population of Medicare beneficiaries 65 years and older in 2011.

^b^ Estimated using design-based Pearson χ^2^ statistics accounting for sampling design and nonresponse.

^c^ Includes American Indian or Alaska Native, Asian, and Native Hawaiian or other Pacific Islander.

^d^ Measured using the 2-item Patient Health Questionnaire.

### Food Insecurity and Change in Cognitive Function

Food insecurity was not associated with baseline cognitive function scores or changes in immediate or delayed recall but was associated with a faster decline in executive function (Table 2). Over time, immediate and delayed recall scores but not executive function scores significantly declined when adjusting for all covariates: immediate and delayed recall scores decreased by −0.09 (95% CI, −0.12 to −0.06) points and −0.05 (95% CI, −0.10 to −0.003) points each year, respectively. The annual change of cognitive function measures was faster among participants experiencing food insecurity compared with those not experiencing food insecurity. Among older adults with food insecurity, the mean decline in executive function scores each year was −0.04 (95% CI, −0.09 to −0.003) points faster than those without food insecurity in an adjusted model. The differences in mean changes comparing participants with vs without food insecurity were 0.01 (95% CI, −0.05 to 0.08) points per year for immediate memory and −0.01 (95% CI, −0.08 to 0.06) points per year for delayed memory, but they were not statistically significant in adjusted models (*P* = .73 and *P* = .78, respectively). Our sensitivity analysis revealed that the direction and magnitude of the coefficient for food insecurity × time interaction in the executive function model was similar but not statistically significant when using a shorter follow-up period (2013-2019) (eTable 1 in Supplement 1). The findings from marginal structural models were consistent with the results from the mixed-effects models (eTables 2 and 3 in Supplement 1).

**Table 2.** Associations Between Food Insecurity and Cognitive Function in the National Health and Aging Trends Study^a^

Cognitive measure^b^	Model terms	Unadjusted coefficient (95% CI)	*P* value	Adjusted coefficient (95% CI)	*P* value
Immediate memory (possible range, 0-10)	Food insecurity	−0.22 (−0.62 to 0.20)	.30	−0.13 (−0.53 to 0.27)	.51
	Time	−0.11 (−0.12 to −0.10)	<.001	−0.09 (−0.12 to −0.06)	<.001
	Food insecurity × time	0.02 (−0.05 to 0.09)	.56	0.01 (−0.05 to 0.08)	.73
Delayed memory (possible range, 0-10)	Food insecurity	−0.18 (−0.60 to 0.25)	.41	−0.04 (−0.46 to 0.38)	.85
	Time	−0.10 (−0.11 to −0.08)	<.001	−0.05 (−0.10 to −0.003)	.04
	Food insecurity × time	0.01 (−0.06 to 0.08)	.78	−0.01 (−0.08 to 0.06)	.78
Executive function (possible range, 0-5)	Food insecurity	0.12 (−0.13 to 0.38)	.34	0.22 (−0.04 to 0.48)	.09
	Time	−0.001 (−0.01 to 0.01)	.82	0.01 (−0.02 to 0.03)	.63
	Food insecurity × time	−0.04 (−0.07 to 0.003)	.07	−0.04 (−0.09 to −0.003)	.04

^a^ Includes 3014 participants from 2013 to 2020. Coefficients obtained from linear mixed-effects models, comparing food insecurity vs no food insecurity. The models included the main effects of age, sex, income, race and ethnicity, marital status, educational level, depressive symptoms, social isolation, body mass index, and disability as well as interactions with time for race and ethnicity, educational level, and age at baseline. Analytic weights were applied each year to account for complex sampling design and nonresponse.

^b^ Higher scores indicate better performance; lower scores indicate worse performance.

## Discussion

This cohort study found that food insecurity was associated with a faster decline in executive function among a nationally representative sample of community-dwelling older adults, but not with faster declines in immediate or delayed memory function. Both food insecurity and cognitive function data were assessed longitudinally to incorporate changes in food insecurity and cognitive performance.

This study expands the literature by testing our hypothesis among a nationally representative sample of US older adults. In a regional study,^15^ over a 2-year follow-up, baseline food insecurity in late life was associated with declines in executive function but not memory among 597 Puerto Rican participants aged 45 to 75 years. The association may be explained by 4 pathways: allostatic load, unhealthy eating patterns, poor disease management, or a lack of cognitive reserve. First, food insecurity indicating financial strain is associated with higher levels of stress.^9,32,33^ Chronic and repeated exposure to psychosocial stress can lead to dysregulation in inflammatory, cardiovascular, and metabolic systems, and the pathophysiological allostatic states consequently result in cognitive decline as well as other chronic diseases related to cognitive declines such as hypertension and diabetes.^34,35,36,37,38^ In particular, exposure to stress can affect the prefrontal cortex region of the brain and the affected brain region may disrupt cognitive processes and executive function.^39^ Furthermore, food insecurity is associated with decreased consumption of vegetables and fruit, which may slow decline in cognitive function.^40,41,42^ Individuals with food insecurity are more likely to have low diabetes medication adherence; uncontrolled diabetes is associated with a faster decline in cognitive function.^43,44^ Additionally, food insecurity is associated with less moderate to vigorous physical activity.^45^ Physical activity is an important indicator of cognitive reserve referring to resilience against changes in brain structure.^46^ Participants with food insecurity may be more susceptible to cognitive decline and showed a faster decline in executive function in the present study.

We observed a decline of 0.28 points in executive function measured by the clock drawing test over the study period among individuals with reported food insecurity compared with those without food insecurity. The cutoff score of the test to detect dementia is 1 on a 5-point scale (1.5 SDs below the mean).^47,48^ The effect of the association between food insecurity and executive function is small, but subtle decline can indicate preclinical dementia^49^ and further cognitive decline in multiple domains.^50^ The clock drawing test is a useful tool to detect dementia early and monitor cognitive change over time,^51^ and it may be more relevant to primary care settings than a specialized care context.^52^ The association between food insecurity and executive function should be paid attention, since food insecurity disproportionally affects racial and ethnic minority and low-income populations, and the disparity is preventable.

Interestingly, this study found that immediate and delayed memory scores but not executive function scores significantly declined with time, and food insecurity was associated with a faster decline in executive function but not memory scores. This finding may suggest that age-related cognitive declines and food insecurity–related cognitive declines may have different effects across cognitive domains. A meta-analysis^53^ suggested that psychosocial stress, a major pathway of food insecurity to cognitive decline, may have no or little association with episodic memory but have an association with executive function. Food insecurity may have associations with executive function than episodic memory. Accelerated declines in executive function associated with food insecurity, not a part of normal cognitive aging, are especially concerning. This is because executive function encompasses a wide range of cognitive processes that allow people to engage in independent and purposive behaviors.^54,55^ Compared with memory, executive function was a stronger factor associated with physical functioning.^56^ Additionally, the higher health care expenditures, even at the early stage of cognitive decline, were largely attributed to executive dysfunction.^57^ Alternatively, it is possible that we found the association of food insecurity with executive function declines because the measure used in this study captures food insecurity due to limited physical functioning, which is associated with executive function. However, we controlled for baseline physical functioning to minimize the confounding bias.

The association between food insecurity and change in executive function was absent after excluding 2020 data in sensitivity analyses. This may be due to duration of follow-up, enlarged disparities in cognition by food insecurity status during the COVID-19, or a different survey administration mode. First, change in cognitive performance measures among community-dwelling older adults without dementia can be slow. Follow-up of participants is required for a long enough period to detect the small change over time. Adding 1 more year of data might allow us to detect the significant change. Alternatively, difference in cognition by food insecurity status could be larger in 2020, the last year cognitive data were collected during the pandemic. During the pandemic, restricted social interaction, higher unemployment rate leading to lower income, higher stress, less cognitive activities, and limited opportunity for physical activities might have indirectly or directly affected cognitive aging. Individuals with food insecurity were likely to have fewer resources to compensate the challenges so that they were exposed to greater risk of cognitive declines. Additionally, different results after excluding 2020 cognition data might be related to altered survey administration mode, but differences in cognitive performance by survey modalities are very small,^20,58^ especially for individuals without cognitive impairment.

This study estimated that baseline prevalence of food insecurity was 2.5% among community-dwelling Medicare beneficiaries 65 years and older. The estimate was smaller than that from another report.^2^ In 2020, prevalence of food insecurity was 6.8% and prevalence of very low food security was 2.6% among participants 60 years and older in the Current Population Survey, which is a nationally representative survey.^2^ The difference in the estimates can be explained by different measures of food insecurity. The food insecurity measure used in our study captured actual experiences while another food insecurity measure, the US Household Food Security Survey Module applied in the Current Population Survey, assessed both worry and actual food insecurity experiences. Another difference in the measures is that we asked about food insecurity experiences in relation not only to financial strain but also lack of social support and physical function, while the latter instrument focused on food insecurity due to financial constraint. This may suggest that the food insecurity measure used in our study captures more severe cases of food insecurity (very low food security). More severe food insecurity may induce even higher levels of stress and adverse health impacts.^59^

### Limitations

This study has several limitations. Due to the nature of observational studies, we cannot establish the causality between food insecurity and cognitive decline. Considerable missing income data were imputed to minimize risk of bias such as selection bias, but this may result in another bias if the missing-at-random assumption is violated. Additionally, we did not correct *P* values for fitting multiple models; thus, the findings on statistical significance should be interpreted with caution.

## Conclusions

The findings of this cohort study suggest that food insecurity in late life was associated with accelerated declines in executive function that is necessary for daily activities. Clinicians should be aware that older adults reporting food insecurity are at higher risk of faster cognitive decline that can be an early mark of cognitive impairment. The adverse effects of food insecurity on cognitive health call for interventions and policies incorporating a variety of approaches such as addressing food quality, food preferences, transportation, food preparation, and food purchase to eliminate food insecurity. Other interventions to delay or prevent cognitive decline such as exercise, stress management, or social groups can be considered for people reporting food insecurity or those at higher risk of food insecurity.

## Notes

Supplement 1. **eMethods.** Adjustment Variables **eFigure.** A Directed Acyclic Graph to Determine Potential Confounders **eTable 1.** Sensitivity Analysis: Conditional Associations Between Food Insecurity With Cognitive Function, National Health and Aging Trends Study, 2013-2019 (n = 3014) **eTable 2.** Sensitivity Analysis: Marginal Associations Between Food Insecurity With Cognitive Function, National Health and Aging Trends Study, 2013-2020 (n = 3015) **eTable 3.** Sensitivity Analysis: Marginal Associations Between Food Insecurity With Cognitive Function, National Health and Aging Trends Study, 2013-2019 (n = 3015)
eReferences

Supplement 2.
Data Sharing Statement
