# Supplementary material for: Food Insecurity and Cognitive Trajectories in Community-Dwelling Medicare Beneficiaries 65 Years and Older
Source: JAMA Netw Open. 2023 Mar 24;6(3):e234674. doi: 10.1001/jamanetworkopen.2023.4674 (PMC10313147; doi:10.1001/jamanetworkopen.2023.4674)
Supplement: Supplement 1. — eMethods. Adjustment Variables eFigure. A Directed Acyclic Graph to Determine Potential Confounders eTable 1. Sensitivity Analysis: Conditional Associations Between Food Insecurity With Cognitive Function, National Health and Aging Trends Study, 2013-2019 (n = 3014) eTable 2. Sensitivity Analysis: Marginal Associations Between Food Insecurity With Cognitive Function, National Health and Aging Trends Study, 2013-2020 (n = 3015) eTable 3. Sensitivity Analysis: Marginal Associations Between Food Insecurity With Cognitive Function, National Health and Aging Trends Study, 2013-2019 (n = 3015) eReferences [file jamanetwopen-e234674-s001.pdf]

# Supplementary Online Content

Kim B, Samuel LJ, Thorpe RJ Jr, Crews DC, Szanton SL. Food insecurity and cognitive trajectories in community-dwelling Medicare beneficiaries 65 years and older. *JAMA Netw Open*. 2023;6(3):e234674.
doi:10.1001/jamanetworkopen.2023.4674

**eMethods.** Adjustment Variables
**eFigure.** A Directed Acyclic Graph to Determine Potential Confounders
**eTable 1.** Sensitivity Analysis: Conditional Associations Between Food Insecurity With Cognitive Function, National Health and Aging Trends Study, 2013-2019 (n = 3014)
**eTable 2.** Sensitivity Analysis: Marginal Associations Between Food Insecurity With Cognitive Function, National Health and Aging Trends Study, 2013-2020 (n = 3015)
**eTable 3.** Sensitivity Analysis: Marginal Associations Between Food Insecurity With Cognitive Function, National Health and Aging Trends Study, 2013-2019 (n = 3015)
**eReferences**

This supplementary material has been provided by the authors to give readers additional information about their work.

© 2023 Kim B et al. *JAMA Network Open*.

**eMethods.** Adjustment Variables

Additional variables included as potential confounders in these analyses were educational attainment [less than high school (referent), high school, more than high school], sex [female, male (referent)], race and ethnicity [White (referent), Black, American Indian or Alaska Native, Asian, and Native Hawaiian or other Pacific Islander, Hispanic], age, depression, functional disability, and social isolation scores at baseline. Presence of depressive symptoms were classified based on scores ≥ 3 on the Patient Health Questionnaire (PHQ-2).[1] Disability refers to receiving assistance for bathing, eating, dressing, toileting, getting out of bed, or walking inside the home; or not done the activities in the past month.[2] The total number of activities with functional disability (possible range 0-6) was categorized as none, one to two, or three to six.[2] Social isolation was measured using a multi-domain typology.[3] Participants received one point (possible range 0-4) for each of the following items: living with at least one other person, having two or more people to talk to about important matters in the last year, attending religious services in the last month, and participating in other organized activities in the last month. As in prior work, participants with isolation scores of 0, 1, or ≥ 2 were classified as severely socially isolated, socially isolated, or socially integrated, respectively.[3] We additionally adjusted for marital status, income [1st tercile: <$18,000 (referent), 2nd tercile: $18,001-$41,000, 3rd tercile: > $41,000], body mass index [underweight (referent), normal weight, overweight, obesity], and residential area [metropolitan (referent), non-metropolitan]. These additional variables were included as a time varying variable because they were not likely on the casual pathways over the study period (i.e., exposure status in 2012 does not affect covariates in 2013). Income was reported in 2011, 2013, 2015, 2017, and 2019, and we assumed stable income from the prior year. Marital status and body mass index were measured between 2012 to 2019 and residential

© 2023 Kim B et al. *JAMA Network Open*.

area data were extracted from 2012 to 2018 data. Year since study enrollment was also included in these analyses as a time metric.

© 2023 Kim B et al. *JAMA Network Open*.

**eFigure.** A Directed Acyclic Graph to Determine Potential Confounders

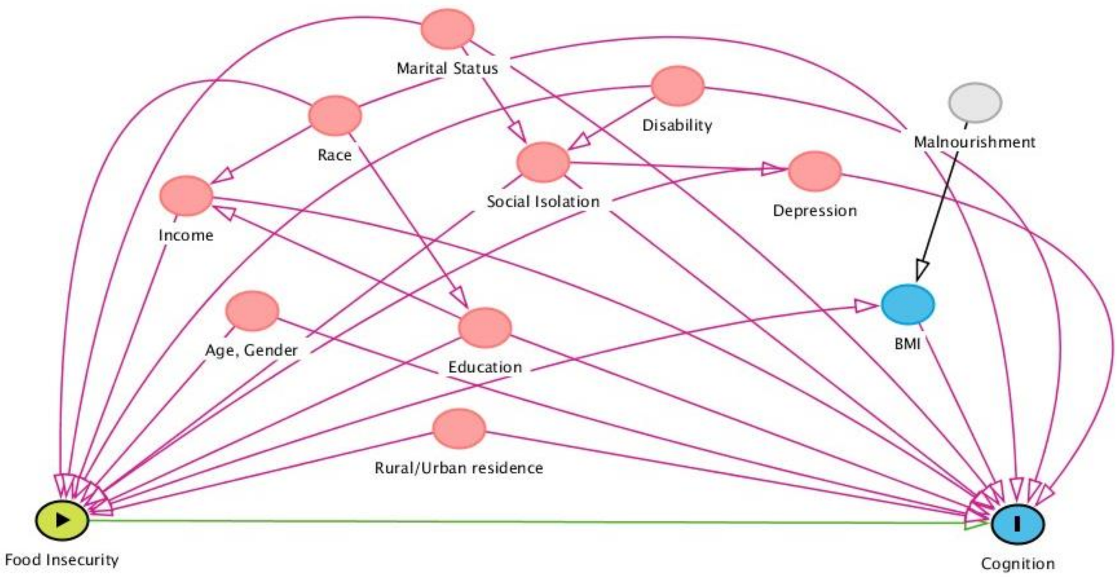


Note: A directed Acyclic Graph was constructed to determine minimal sets of potential confounders to adjust for estimating the association between food insecurity and cognition. *For mixed-effect models with 1-year lagged analysis*, variables that are not likely on the causal pathways (i.e., exposure in previous year does not influence covariables in the next year), including income, marital status, rural/urban residence, and body mass index, were included into the models as time-varying variables. Other variables, including age, sex, educational level, race and ethnicity, social isolation, disability, and depression were included as time-fixed variables because they were assumed to be stable over time or likely on the causal pathways over time. *For marginal structural models*, we included income, marital status, rural/urban residence, body mass index, social isolation, disability, and depression as time-varying variables. Baseline values of age, sex, educational level, and race and ethnicity were included as time-fixed variables.

© 2023 Kim B et al. *JAMA Network Open*.

**eTable 1.** Sensitivity Analysis: Conditional Associations Between Food Insecurity With Cognitive Function, National Health and Aging Trends Study, 2013-2019 (n = 3014)

| Cognitive Measure | Model Terms | Unadjusted coefficient (95% CI) | *P* Value | Adjusted coefficient (95% CI) | *P* Value |
|---|---|---|---|---|---|
| **Immediate memory** (possible range 0-10) | Food insecurity | -0.26 (-0.65 – 0.13) | .19 | -0.06 (-0.09 – -0.02) | .005 |
| | Time | -0.09 (-0.10 – -0.08) | <.001 | -0.18 (-0.58 – 0.21) | .36 |
| | Food insecurity × Time | 0.03 (-0.03 – 0.10) | .35 | 0.02 (-0.05 – 0.09) | .52 |
| **Delayed memory** (possible range 0-10) | Food insecurity | -0.03 (-0.45 – 0.40) | .89 | 0.13 (-0.28 – 0.54) | .61 |
| | Time | -0.09 (-0.11 – -0.08) | <.001 | -0.03 (-0.09 – 0.02) | .20 |
| | Food insecurity × Time | -0.02 (-0.09 – 0.06) | .67 | -0.04 (-0.11 – 0.03) | .25 |
| **Executive function** (possible range 0-5) | Food insecurity | 0.04 (-0.25 – 0.34) | .77 | 0.16 (-0.14 – 0.45) | .29 |
| | Time | -0.01 (-0.01 – 0.00) | .13 | 0.01 (-0.01 – 0.04) | .33 |
| | Food insecurity × Time | -0.02 (-0.07 – 0.03) | .42 | -0.03 (-0.08 – 0.02) | .22 |

Note: Coefficients obtained from linear mixed-effects models, comparing food insecurity versus no food insecurity. The models included main effects of age, sex, income, race and ethnicity, marital status, educational level, depressive symptoms, social isolation, body mass index and disability as well as interactions with time for race and ethnicity, educational level and age at baseline. We applied analytic weights each year to take into account complex sampling design and nonresponse.

© 2023 Kim B et al. *JAMA Network Open*.

**eTable 2.** Sensitivity Analysis: Marginal Associations Between Food Insecurity With Cognitive Function, National Health and Aging Trends Study, 2013-2020 (n = 3015)

| Cognitive Measure | Model Terms | Coefficient (95% CI) | *P* Value |
|---|---|---|---|
| **Immediate memory** (possible range 0-10) | Food insecurity | -0.20 (-0.56 – 0.16) | .28 |
| | Time | -0.13 (-0.14 – -0.12) | <.001 |
| | Food insecurity × Time | 0.02 (-0.04 – 0.08) | .48 |
| **Delayed memory** (possible range 0-10) | Food insecurity | 0.04 (-0.43 – 0.52) | .86 |
| | Time | -0.11 (-0.12 – -0.10) | <.001 |
| | Food insecurity × Time | -0.01 (-0.08 – 0.06) | .78 |
| **Executive function** (possible range 0-5) | Food insecurity | 0.25 (-0.06 – 0.56) | .12 |
| | Time | -0.01 (-0.02 – 0.00) | .004 |
| | Food insecurity × Time | -0.05 (-0.10 – -0.01) | .03 |

Note: Coefficients obtained from marginal structural models, comparing food insecurity versus no food insecurity. To minimize over-adjustment of potential confounders or controlling for variables on the causal pathways, we performed marginal structural models with stabilized inverse propensity weights as sensitivity analysis. The conditional probability of reporting food insecurity was estimated using logistic regression based on time-varying potential confounders (i.e., income, marital status, rural/urban residence, body mass index, social isolation, disability, and depression) and baseline confounders (i.e., age, sex, educational level, and race and ethnicity). Inverse propensity weights were separately generated for individuals with or without food insecurity. The inverse propensity weights were multiplied by analytic weights provided by NHATS to take into account nonresponse. Robust standard errors were used to construct confidential intervals.

© 2023 Kim B et al. *JAMA Network Open*.

**eTable 3.** Sensitivity Analysis: Marginal Associations Between Food Insecurity With Cognitive Function, National Health and Aging Trends Study, 2013-2019 (n = 3015)

| Cognitive Measure | Model Terms | Coefficient (95% CI) | *P* Value |
|---|---|---|---|
| **Immediate memory** (possible range 0-10) | Food insecurity | -0.23 (-0.62 – 0.16) | .25 |
| | Time | -0.11 (-0.12 – -0.09) | <.001 |
| | Food insecurity × Time | 0.03 (-0.04 – 0.09) | .42 |
| **Delayed memory** (possible range 0-10) | Food insecurity | 0.22 (-0.13 – 0.75) | .41 |
| | Time | -0.11 (-0.12 – -0.10) | <.001 |
| | Food insecurity × Time | -0.04 (-0.12 – 0.04) | .34 |
| **Executive function** (possible range 0-5) | Food insecurity | 0.25 (-0.10 – 0.59) | .16 |
| | Time | -0.01 (-0.02 – -0.01) | <.001 |
| | Food insecurity × Time | -0.05 (-0.11 – 0.01) | .08 |

Note: Coefficients obtained from marginal structural models, comparing food insecurity versus no food insecurity. To minimize over-adjustment of potential confounders or controlling for variables on the causal pathways, we performed marginal structural models with stabilized inverse propensity weights as sensitivity analysis. The conditional probability of reporting food insecurity was estimated using logistic regression based on time-varying potential confounders (i.e., income, marital status, rural/urban residence, body mass index, social isolation, disability, and depression) and baseline confounders (i.e., age, sex, educational level, and race and ethnicity). Inverse propensity weights were separately generated for individuals with or without food insecurity. The inverse propensity weights were multiplied by analytic weights provided by NHATS to take into account nonresponse. Robust standard errors were used to construct confidential intervals.

© 2023 Kim B et al. *JAMA Network Open*.

**eReferences**


1. Löwe B, Kroenke K, Gräfe K. Detecting and monitoring depression with a two-item questionnaire (PHQ-2). *J Psychosom Res*. 2005;58(2):163-171. doi:10.1016/j.jpsychores.2004.09.006
2. Ankuda CK, Fogel J, Kelley AS, Byhoff E. Patterns of material hardship and food insecurity among older adults during the COVID-19 pandemic. *J Gen Intern Med*. 2021;36(11):3639-3641. doi:10.1007/s11606-021-06905-3
3. Cudjoe TKM, Roth DL, Szanton SL, Wolff JL, Boyd CM, Thorpe RJ. The epidemiology of social isolation: National Health and Aging Trends Study. *J Gerontol B Psychol Sci Soc Sci*. 2020;75(1):107-113. doi:10.1093/geronb/gby037


© 2023 Kim B et al. *JAMA Network Open*.
