# Supplementary material for: Food Insecurity and Cognitive Trajectories in Community-Dwelling Medicare Beneficiaries 65 Years and Older
Source: JAMA Netw Open. 2023 Mar 24;6(3):e234674. doi: 10.1001/jamanetworkopen.2023.4674 (PMC10313147; doi:10.1001/jamanetworkopen.2023.4674)
Supplement: Supplement 2. — Data Sharing Statement [file jamanetwopen-e234674-s002.pdf]

Data Sharing Statement

Kim. Food Insecurity and Cognitive Trajectories in Community-Dwelling Medicare Beneficiaries 65 Years and Older. *JAMA Netw Open*. Published March 24, 2023. doi:10.1001/jamanetworkopen.2023.4674

Data
Data available: No

Additional Information
Explanation for why data not available: NHATS datasets are already publicly available.
